# Transcriptome Analysis Reveals Distinct Responses to Physiologic *versus* Toxic Manganese Exposure in Human Neuroblastoma Cells

**DOI:** 10.3389/fgene.2019.00676

**Published:** 2019-07-24

**Authors:** Jolyn Fernandes, Joshua D. Chandler, Loukia N. Lili, Karan Uppal, Xin Hu, Li Hao, Young-Mi Go, Dean P. Jones

**Affiliations:** Division of Pulmonary, Allergy, Critical Care and Sleep Medicine, Department of Medicine, Emory University, Atlanta, GA, United States

**Keywords:** dose-response, cell transcriptomics, adaptive response, neurotoxicity, metals

## Abstract

Manganese (Mn) is an essential trace element, which also causes neurotoxicity in exposed occupational workers. Mn causes mitochondrial toxicity; however, little is known about transcriptional responses discriminated by physiological and toxicological levels of Mn. Identification of such mechanisms could provide means to evaluate risk of Mn toxicity and also potential avenues to protect against adverse effects. To study the Mn dose-response effects on transcription, analyzed by RNA-Seq, we used human SH-SY5Y neuroblastoma cells exposed for 5 h to Mn (0 to 100 μM), a time point where no immediate cell death occurred at any of the doses. Results showed widespread effects on abundance of protein-coding genes for metabolism of reactive oxygen species, energy sensing, glycolysis, and protein homeostasis including the unfolded protein response and transcriptional regulation. Exposure to a concentration (10 μM Mn for 5 h) that did not result in cell death after 24-h increased abundance of differentially expressed genes (DEGs) in the protein secretion pathway that function in protein trafficking and cellular homeostasis. These include *BET1* (Golgi vesicular membrane-trafficking protein), *ADAM10* (ADAM metallopeptidase domain 10), and *ARFGAP3* (ADP-ribosylation factor GTPase-activating protein 3). In contrast, 5-h exposure to 100 μM Mn, a concentration that caused cell death after 24 h, increased abundance of DEGs for components of the mitochondrial oxidative phosphorylation pathway. Integrated pathway analysis results showed that protein secretion gene set was associated with amino acid metabolites in response to 10 μM Mn, while oxidative phosphorylation gene set was associated with energy, lipid, and neurotransmitter metabolites at 100 μM Mn. These results show that differential effects of Mn occur at a concentration which does not cause subsequent cell death compared to a concentration that causes subsequent cell death. If these responses translate to effects on the secretory pathway and mitochondrial functions *in vivo*, differential activities of these systems could provide a sensitive basis to discriminate sub-toxic and toxic environmental and occupational Mn exposures.

## Introduction

Manganese (Mn) is an essential element in humans that is required for antioxidant defense and structural stability of proteins (Christianson, 1997; Aguirre and Culotta, 2012; Avila et al., 2013; Smith et al., 2017). Variation in Mn has great impact on pathological outcomes indicated by association of high Mn with low IQ scores (Wasserman et al., 2006; Bouchard et al., 2011) and cognitive decline (Menezes-Filho et al., 2011; Kornblith et al., 2017), along with occupational exposures leading to neurological disorders (Lucchini et al., 1995; Bouchard et al., 2005; Cowan et al., 2009). Because of shared transport systems between Mn and many essential metal ions such as Fe, Zn, Cu, and Ca (Rossander-Hulten et al., 1991; Guerinot, 2000; Pinsino et al., 2011; Mercadante et al., 2016; Smith et al., 2017), Mn can potentially hijack gene regulation and transcriptional control by these other metals, rendering the cell susceptible to Mn toxicity. Focusing only on the adverse outcomes of Mn, however, overlooks the need for mechanistic understanding of adaptive responses to maintain cellular homeostasis and resist toxic effects.

In recent years, studies have shown that microarray analysis and deep-sequencing technologies such as RNA-Seq analysis can be exploited to determine adaptive pathways, which regulate responses to stressors (Pillai et al., 2014; Currier et al., 2016; Zheng et al., 2018). Previous microarray studies linking toxicity and adaptive transcriptomic and proteomic responses in green algae, *Chlamydomonas reinhardtii*, exposed to silver showed that the cells initiate a defense response to combat oxidative stress and eliminate silver *via* efflux transporters (Pillai et al., 2014). Another microarray study used a similar strategy to identify enrichment in p53 signaling and NRF2-related genes in adaptive and adverse responses to zinc in human bronchial epithelial cells (Currier et al., 2016). For Mn exposures, there is no information on Mn dose-response effects on the gene expression profile with a graded model from non-toxic to toxic exposures.

Prior research with a human neuroblastoma (SH-SY5Y) cell line showed that controlled 5-h exposure to establish cellular Mn content over the range found in normal human brain and human brain from individuals with Mn-dependent pathology resulted in distinct physiologic and toxic responses (Fernandes et al., 2017). As Mn dose was increased, increased oxidative stress was represented by decreased cellular thiols, increased mitochondrial H_2_O_2_ production, and biphasic change in mitochondrial respiration (Fernandes et al., 2017). Respiration increased to a maximum at the highest dose supporting cell survival (10 μM MnCl_2_) after 24 h and decreased at concentrations (≥50 μM MnCl_2_) resulting in cell death after 24 h (Fernandes et al., 2017; Fernandes et al., 2019). In these studies, no cell death was detected at 5 h for any Mn concentration studied. However, substantial cell death was observed after a 24-h recovery period in cells treated with 50 or 100 μM Mn for 5 h while no significant cell death was observed after 24 h recovery in Mn concentrations ≤ 10, thus demonstrating ≥ 50 μM Mn as a toxic dose (Fernandes et al., 2019). Subsequent metabolomics studies using this model further allowed discrimination of adaptive responses to physiologic variation of Mn from toxic responses resulting in subsequent cell death after 24 h (Fernandes et al., 2019). Metabolomics further showed increases in neuroprotective amino acid metabolites (creatine, phosphocreatine, phosphoserine) after 5 h at a non-toxic 10 μM Mn while 5-h exposure to a concentration that subsequently caused cell death resulted in decreases in energy and fatty acid metabolites (hexose-1,6 bisphosphate, acyl carnitines).

Based upon these previous results, in the present study, we applied RNA-Seq analysis to distinguish transcriptome responses at 5 h under physiologic Mn exposures (0, 1, 5, 10 μM; no cell death after 24 h) and toxic Mn exposures (50 and 100 μM; cell death after 24 h). Results showed widespread effects of Mn on gene network and candidate genes over the entire dose-response range and suggested that variations in gene sets linked to secretory and mitochondrial functions could provide a basis to discriminate adaptive and toxic responses to environmental and occupational Mn exposures.

## Materials and Methods

### Cell Culture and Manganese Dose-Dependent Exposure

SH-SY5Y, human neuroblastoma cell line, obtained from the American Type Culture Collection (ATCC-Manassas, VA) was cultured and exposed to Mn dose as previously described (**Supplementary Datasheet 1**) (Fernandes et al., 2017; Fernandes et al., 2018; Fernandes et al., 2019). Briefly, cells were cultured in Dulbecco’s modified Eagle medium/Ham’s F12 medium and, at 80% confluency, were treated with Mn (0, 1, 5, 10, 50, 100 μM as MnCl_2_; Sigma-Aldrich, St. Louis, MO) for 5 h. The 5-h time point was chosen to correlate transcriptomic changes with cellular Mn concentration comprising physiological (≤10 μM Mn treatment) to pathophysiological Mn levels (≥50 μM Mn treatment) that was optimized previously (Fernandes et al., 2017) based on the recommended dosage of treatment and accumulation of cellular Mn relevant to *in vivo* levels from human brain and *in vitro* studies (Csaszma et al., 2003; Bowman and Aschner, 2014; Kumar et al., 2015; Fernandes et al., 2017). Briefly, cellular Mn content for this experimental design was reported previously (μM as MnCl_2,_ ng Mn/mg protein ± SEM): 0, 6.4 ± 1.0; 1, 12.0 ± 0.7; 5, 12.7 ± 1.8; 10, 15.7 ± 1.1; 50, 36.8 ± 1.8; and 100, 49.2 ± 0.5 (Fernandes et al., 2017).

#### RNA Extraction

For each of the six experimental conditions of Mn concentration, a total of three biological replicate samples were used for RNA isolation and quantification. Total RNA was extracted and isolated using miRNeasy Mini Kit (Qiagen). Quality control analyses were performed for each of the samples and were acceptable for downstream sequencing if they met the following criteria: 260/280 ratio (nucleic acid purity) > 2 and RIN score (RNA integrity number) > 8.5 (Schroeder et al., 2006).

#### RNA-Seq Data Processing and Analysis

Initial RNA samples were poly(A) selected to eliminate rRNA, and the cDNA library was prepared using random hexamers and further ribosomal depletion. The platform used for sequencing was the Illumina HiSeq 2500 that ran eight technical replicates (four forward and four reverse) for each of the 18 samples in pair-end mode. This resulted in 144 raw.fastq files that were further exploited in the downstream data analysis and interpretation. Initial data quality control was performed using the FastQC tool (Babraham Bioinformatics, version 0.11.4; Andrews, 2010). Following quality control and assurance of technically sound sequencing files, the trimming of the low-quality read ends was executed to ensure a Phred score > 30. The trimming was completed with the TrimGalore! tool (Babraham Bioinformatics, version 0.4.1; Krueger). The trimmed reads were then aligned to the human genome (December 2013, hg38) with the STAR alignment algorithm, version 2.5.2a (Dobin et al., 2013), and by using the RefSeq annotation of genes downloaded from the UCSC genome browser (http://genome.ucsc.edu/index.html). The read counts were then calculated using HTSeq, version 0.6.1 (Anders et al., 2015) with the default union mode. In total, 39,287 transcripts were detected and for further downstream statistical analysis, the read counts were normalized by the variance stabilization transformation as implemented in the DESeq2 R/Bioconductor package (Love et al., 2014). The datasets were filtered and multiple transcripts that belong to the same gene name were averaged, which resulted in 15,066 transcripts with unique identifiers used for further downstream analysis.

#### Gene Set Pathway Enrichment Analysis

Gene Set Enrichment Analysis (GSEA) (Subramanian et al., 2005) was used to identify groups of genes enriched by Mn dose. The GSEA analysis tool (version 2.0) was downloaded from the Broad Institute website (http://www.broadinstitute.org/gsea/index.jsp). Hallmark gene sets from Molecular Signatures Database (MSigDB) were used to compute overlaps in the current analysis (Broad Institute’s MSigDB). Sets containing 15–500 genes were analyzed. The entire list of 15,066 genes was pre-ranked by the ß coefficient obtained from linear regression against cellular Mn concentration prior to GSEA. To determine adaptive (0 *vs*. 10 μM Mn) and toxic responses (0 *vs.* 100 μM Mn), categorical analysis was adopted wherein the signal-to-noise ranking metric (default method) was used to pre-ranked genes in GSEA analysis.

#### Statistics and Bioinformatics

The normalized filtered data set was tested for correlation with percent-scaled cellular Mn concentration using a linear regression model (lmreg, *P* ≤ 0.05). The resulting ß coefficient was used to pre-ranked genes for pathway enrichment analysis as stated above. Gene sets significant at false discovery rate (FDR)‐adjusted *q* < 0.2 and containing at least one differentially expressed gene (*P* < 0.05 by lmreg and VIP > 2.0 by PLS regression) in the leading edge were then analyzed. Two-way hierarchical clustering analysis was performed using the hclust function in R to visualize and identify clusters of samples and discriminatory transcripts associated with the Mn concentrations. The heatmap shows unsupervised clustering of samples (columns) and transcripts (rows). To identify differentially expressed genes contributing most to separation by Mn treatment groups categorically, PLS-DA (partial least-square discriminant analysis) was performed using MetaboAnalyst (Xia and Wishart, 2016) and PLS-DA function in R package mixOmics (Rohart et al., 2017). Discriminatory genes that showed the most separation of different Mn treatment were selected based on the variable importance for projection (VIP > 1.3) measure.

Gene expression analysis and high-resolution metabolomics (metabolomics dataset under same experimental conditions obtained from Fernandes et al. (2019) was re-purposed for the current study, and no prior analysis was used) were used to identify key metabolites associated with targeted enriched gene sets using the open-source software, *xMWAS* 0.54 (Uppal et al., 2018), which provides data-driven integration, network visualization, clustering, and differential network analysis. The sPLS (sparse partial least squares) canonical correlation network was restricted to a correlation threshold of 0.4 at *P*-value 0.05 with eigenvector centrality. Joint pathway analysis was used to determine enriched metabolic pathways associated with targeted gene sets using MetaboAnalyst 4.0 (Chong et al., 2018) which incorporates the *mummichog* algorithm (Li et al., 2013). Metabolites were annotated using *xMSannotator*, network-based annotation software, with confidence levels 2–3 (medium–highest confidence using HMDB and KEGG databases) (Uppal et al., 2016).

## Results

### Transcriptome-Wide Association Study (TWAS) of Mn

**Figure 1:**
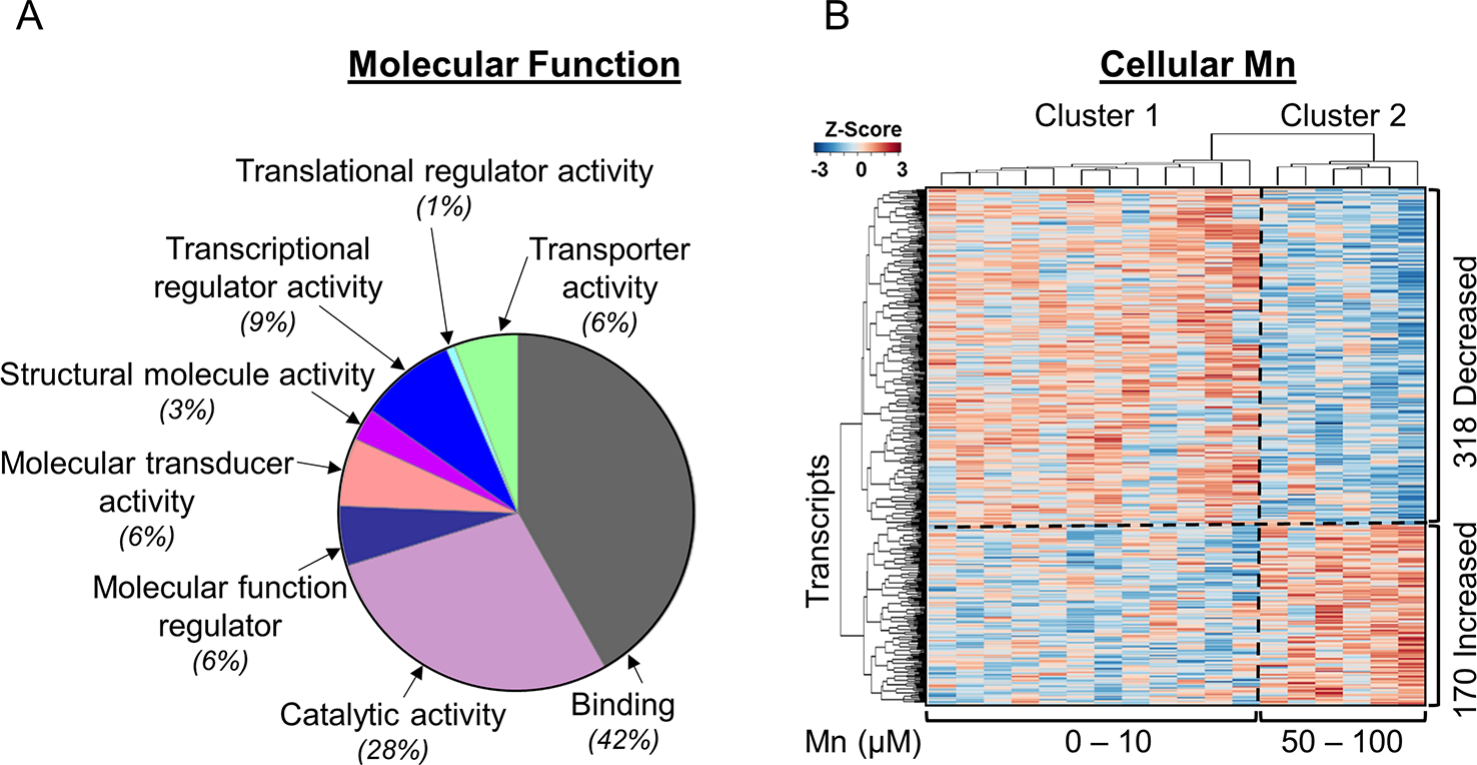
Mn-dependent transcriptome-wide association study and molecular functional classification. **(A)** Molecular functional classification of the 488 discriminatory transcripts by PANTHER database is represented as a pie chart with 42% of the transcripts representing binding function; 28%, catalytic activity; and 9%, transcriptional regulator activity. The rest of the clusters constitute ≤6% of the transcripts in each category. **(B)** The heatmap shows unsupervised clustering of samples (columns) and transcripts (rows), clustered into two major groups representing physiologic and toxic cellular Mn concentration. The clustering is marked by a vertical dotted line. Three hundred eighteen transcripts decreased and 170 transcripts increased with increasing Mn cellular accumulation, which is depicted by the horizontal dotted line. Blue: represents decrease and red: represents increase in transcript expression with increase in cellular Mn. Details on the 488 discriminatory transcripts are provided in the **Supplementary Table S1**. (*n* = 3 per treatment group, *P* < 0.05).

To identify gene expression patterns specific for Mn dose response, we performed a linear regression of 15,066 transcripts obtained by RNA-Seq with cellular Mn concentration. This TWAS showed that expression levels of 3.2% (488/15,066) transcripts were significantly associated with increasing cellular Mn concentration at *P* < 0.05. PLS regression with Mn dose resulted in the same 488 genes significant at VIP > 2. Details on these 488 discriminatory transcripts are provided in **Supplementary Table S1**. To determine molecular functions represented by the 488 differentially expressed genes in the human SH-SY5Y cells, the list of gene names was used as input for protein analysis through evolutionary relationships platform (PANTHER, version 11, http://PANTHERdb.org/; Mi et al., 2017) with *Homo sapiens* as the selected organism. Out of the 488 differentially expressed genes, 275 total function hits were detected. The results are presented as a pie chart (**Figure 1A**) with percent of gene hits against total function hits. A total of 42% of these genes represented binding function, defined by the gene ontology term (GO:0005488) as a selective, non-covalent, and often stoichiometric interaction of a molecule with one or more specific sites on another molecule (Gene Ontology, 2008). While 28% comprised of genes for proteins participating in catalytic activity defined by GO:0003824, other functions included transcription regulator activity (GO:0140110, 9%), molecular transducer activity (GO:0060089, 6%), transporter activity (GO:0005215, 6%), molecular function regulator (GO:0098772, 6%), structural molecule activity (GO:0005198, 3%), and translational regulator activity (GO:0045182, 1%). These results show that even though Mn affected only 3.2% of the transcriptome, the genes represented a wide variety of regulation, transport, and signaling functions. Details on genes included in each molecular function category are provided in **Supplementary Table S1**.

Unsupervised two-way hierarchal clustering results showed that a majority (65%, 318) of the transcripts decreased in abundance while the remainder (35%, 170) increased with increasing Mn (**Figure 1B**). The results also showed two main clusters of samples (top dendrogram) with cluster 1 including cells dosed with 0 to 10 μM Mn that did not cause cell death, termed as physiologic range, and cluster 2 including cells dosed with 50 or 100 μM Mn, a concentration that resulted in subsequent cell death after 24 h, termed as toxic range (**Figure 1B**) (Fernandes et al., 2019).

### Partial Least-Square Discriminant Analysis (PLS-DA) of Mn Dose Response

**Figure 2:**
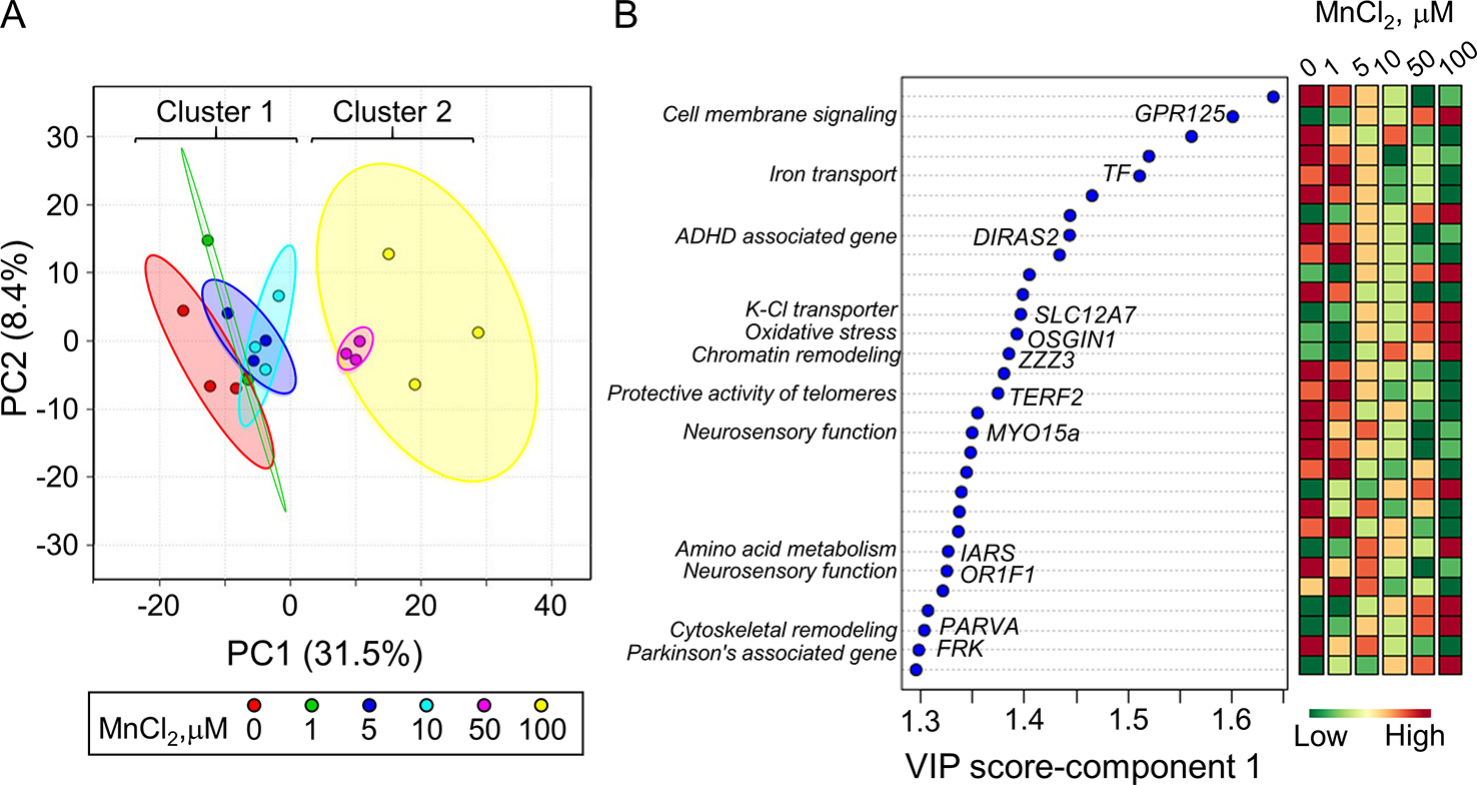
Discriminatory genes contributing to Mn dose-dependent separation as determined by partial least-square discriminant analysis (PLS-DA). **(A)** PLS-DA score plot for transcriptomics data from **Figure 1**, resulting in an Mn dose-dependent separation along principal component 1 (PC1-31.5%) is presented. **(B)** The top 30 discriminatory genes contributing the most to the separation by Mn dose is listed from top to bottom, ranked by their VIP scores (variable importance in projection scores, VIP ≥ 1.3). Changes in expression for each gene at each Mn dose are presented, wherein green: represents decrease and red: represents increase in transcript expression with increase in cellular Mn. The biological function associated with each gene is highlighted. Details on the top-ranked genes are provided in **Table 1**.

PLS-DA was performed to determine top-ranked transcripts from the 488 differentially expressed genes that contributed most to the separation of Mn doses categorically. The score plot showed separation according to Mn dose, wherein 31.5% variation attributed to principal component 1 (PC1) and minimal separation by principal component 2 (PC2) (**Figure 2A**). The physiologic (cluster 1) and toxic (cluster 2) Mn groups were clearly separated by PC1. The variable importance of projection (VIP) score identified the top 30 contributing transcripts (VIP ≥ 1.3), with the priority list ranked from top to bottom (**Figure 2B**). Detailed description of the top-ranked genes is presented in **Table 1**, many of which have unknown functions. Transcripts that increased with Mn dose included genes related to cell membrane signaling (*GPR125*), potassium-chloride transport (*SLC12A7*), oxidative stress (*OSGIN1*), chromatin remodeling (*ZZZ3*), amino acid metabolism (*IARS*), and cytoskeletal remodeling (*PARVA*). The results also showed a number of genes that decreased in an Mn dose-dependent manner which include genes involved in iron transport (*TF*), protective activity of telomeres (*TERF2*), neurosensory function (*MYO15a, OR1F1*), and neurological disease such as ADHD (*DIRAS2*) and Parkinson’s disease (*FRK*). These data show that the transcripts contributing most to the separation according to Mn dose were broadly distributed among biological functions.

**Table 1:** Top-ranked transcripts based on partial least-square discriminant analysis (PLS-DA) of Mn dose response (Top 30 transcripts, VIP ≥ 1.3).

Mn dose effect	Gene name	Gene description (*Homo sapiens*)	VIP score	*P*-value	Literature
***Up***	*GPR125*	Adhesion G protein–coupled receptor A3	1.60	<0.0001	Cell membrane signaling Brain injury (Pickering et al., 2008)
	*TAF5L*	TAF5-like RNA polymerase II, p300/CBP-associated associated factor, 65kDa	1.44	0.0025	-
	*TEX9*	Testis expressed 9	1.40	0.0051	
	*SLC12A7*	Solute carrier family 12	1.40	0.0007	Potassium/chloride transporter (Karadsheh et al., 2004)
	*OSGIN1*	Oxidative stress–induced growth inhibitor 1	1.40	0.0001	Oxidative stress response (Brennan et al., 2017)
	*ZZZ3*	Zinc finger, ZZ-type containing 3	1.38	0.0084	Chromatin remodeling (Mi et al., 2018)
	*ANKRD37*	Ankyrin repeat domain 37	1.34	0.0002	−
	*IARS*	Isoleucyl-tRNA synthetase	1.33	0.0201	Amino acid metabolism; gene mutation associated with intellectual disability (Kopajtich et al., 2016)
	*SNORD116-27*	Small nucleolar RNA, C/D box 116-27	1.31	0.0049	−
	*PARVA*	Parvin, alpha	1.30	0.0126	Cytoskeletal remodeling (Olski et al., 2001)
	*FUT4*	Fucosyltransferase 4 (alpha(1,3)fucosyltransferase, myeloid-specific)	1.30	0.0030	−
***Down***	*LINC00987*	Long intergenic non-protein-coding RNA 987	1.64	0.0001	−
	*SKIDA1*	SKI/DACH domain containing 1	1.56	<0.0001	−
	*PEAR1*	Platelet endothelial aggregation receptor 1	1.52	0.0073	−
	*TF*	Transferrin	1.51	0.0020	Iron transport (Gkouvatsos et al., 2012)
	*A1BG-AS1*	A1BG antisense RNA 1	1.47	0.0059	
	*DIRAS2*	DIRAS family, GTP-binding RAS-like 2	1.44	0.0023	ADHD-associated gene (Reif et al., 2011; Grunewald et al., 2018)
	*C21orf58*	Chromosome 21 open reading frame 58	1.43	0.0004	−
	*CHURC1-FNTB*	CHURC1-FNTB readthrough	1.40	0.0004	−
	*ASGR1*	Asialoglycoprotein receptor 1	1.38	0.0126	−
	*TERF2*	Telomeric repeat–binding factor 2	1.37	0.0041	Protective activity of telomeres (Zhang et al., 2007)
	*KRTAP5-AS1*	KRTAP5-1/KRTAP5-2 antisense RNA 1	1.35	0.0006	−
	*MYO15A*	Myosin XVA	1.35	0.0065	Neurosensory function (Lloyd et al., 2001)
	*TMEM105*	Transmembrane protein 105 (long noncoding RNA)	1.35	0.0055	−
	*FAM154B*	Stabilizer of axonemal microtubules 2	1.34	0.0125	−
	*ALPK3*	Alpha-kinase 3	1.34	0.0107	−
	*CAHM*	Colon adenocarcinoma hypermethylated (non-protein coding)	1.34	0.0018	−
	*OR1F1*	Olfactory receptor, family 1, subfamily F, member 1	1.33	0.0042	Neurosensory function (Malnic et al., 2004)
	*C2orf68*	Chromosome 2 open reading frame 68	1.32	0.0016	−
	*FRK*	Fyn-related Src family tyrosine kinase	1.30	0.0030	Cell cycle, Parkinson’s associated gene (Anneren et al., 2003; Tan et al., 2018)

### Pathway Enrichment Analysis and Associated Differentially Expressed Genes Responsive to Increasing Mn Exposure

**Figure 3:**
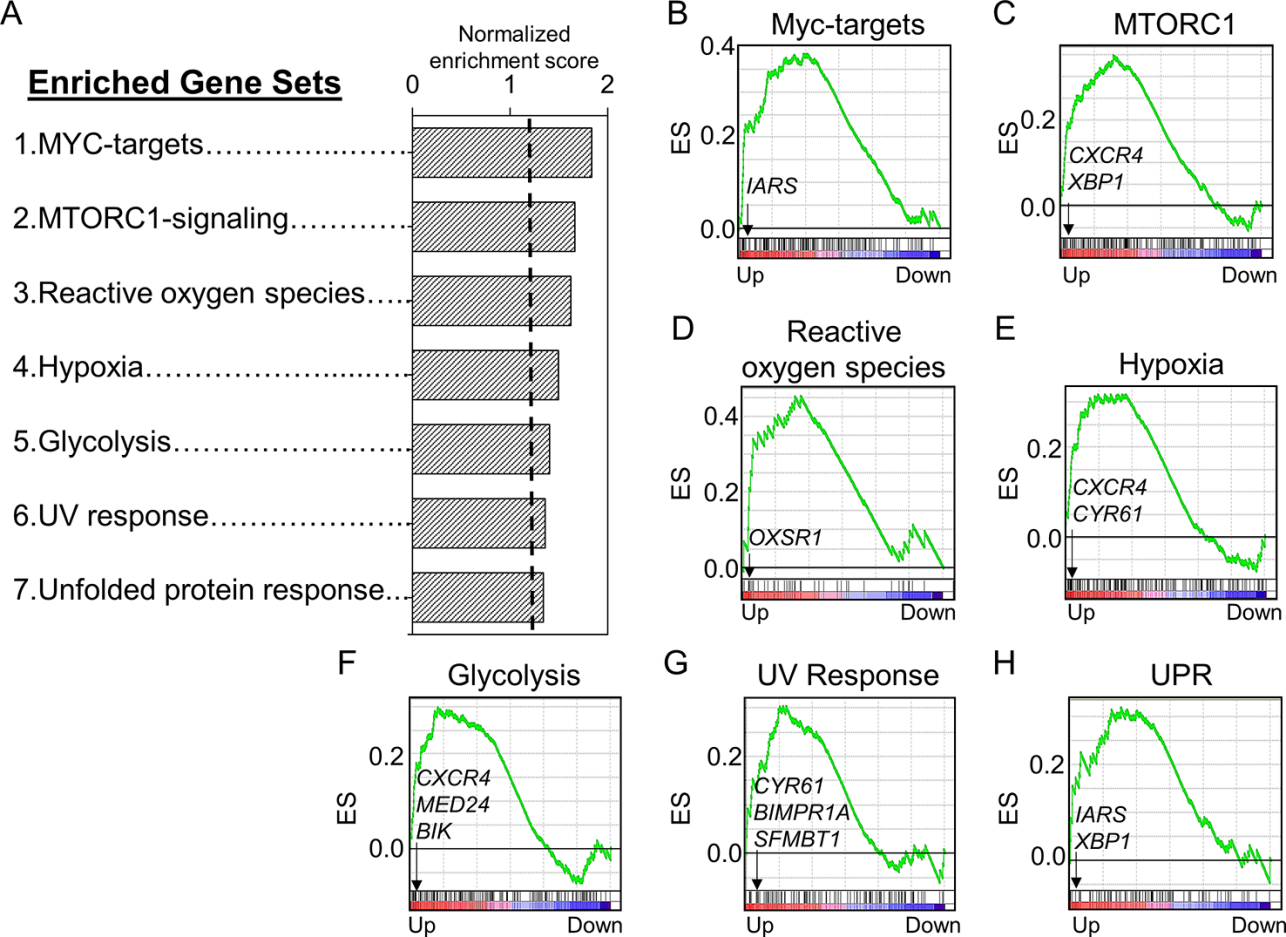
Gene sets and their associated enrichment plots generated by Gene Set Enrichment Analysis (GSEA) of pre-ranked gene expression data. **(A)** A total of seven gene sets enriched by Mn dose at a false discovery rate (FDR) < 0.2 are depicted by their normalized enrichment score ranked from top to bottom. Enrichment plot with enrichment scores (ES) and differentially expressed genes are shown for **(B)** transcriptional regulation—MYC targets, **(C)** nutrient/energy/redox sensing pathway—MTORC signaling, **(D–E)** redox-related pathways—reactive oxygen species and hypoxia, **(F)** energy-related pathway—glycolysis, and **(G–H)** cellular stress response and disruption in protein homeostasis pathways—UV response and unfolded protein response (UPR). The enrichment score is shown as a curve, and the vertical black bars below the plot indicate the position of the gene set–associated leading edge genes, which are mostly grouped in the fraction of up-regulated genes (red) for all the gene sets. Details on the leading edge genes and their respective gene sets are provided in **Supplementary Table S2**. (*P* < 0.05, FDR < 0.2).

We used a pathway enrichment approach to improve understanding of major impacts under increasing Mn exposures. Genes were pre-ranked according to ß coefficient, which was generated using lmreg analysis as previously described, and used as input for GSEA to determine pathways enriched by increasing Mn dose. Results showed that seven gene sets were significantly enriched at an FDR < 0.2, ranked from top to bottom based on their normalized enrichment score (**Figure 3A**). Each of the gene sets had at least one significant gene from the lmreg analysis, as listed in the enrichment plot (**Figures 3B–H**, See**Supplementary Table S1**). Detailed list of all the leading edge genes enriched in each of the seven gene sets is provided in **Supplementary Table S2**. Of the seven gene sets, transcriptional regulation included MYC targets (*IARS*), which was also the topmost enriched pathway by Mn dose (**Figure 3B**). Nutrient, energy, and redox sensing pathway was represented by MTORC1-signaling (*CXCR4, XBP1*) gene set (**Figure 3C**), while glycolysis gene set (*CXCR4*, *MED24*, and *BIK*) represented energy related pathway (**Figure 3F**). Redox-related pathways included reactive oxygen species (*OXSR1*) and hypoxia (*CXCR4*, *CYR61*) gene sets (**Figures 3D, E**). Cellular stress and disruption in protein homeostasis related pathways included UV response (*CYR61*, *BMPR1A*, and *SFMBT1*) and unfolded protein response (UPR) (*IARS*, *XBP1*) gene sets (**Figures 3G, H**). The majority of genes enriched in the seven gene sets were upregulated with increase in Mn dose. Hence, Mn dose-dependent transcriptomic response was enriched in redox signaling, cellular stress, disruption in protein homeostasis, transcriptional regulation, and energy related pathways.

**Figure 4:**
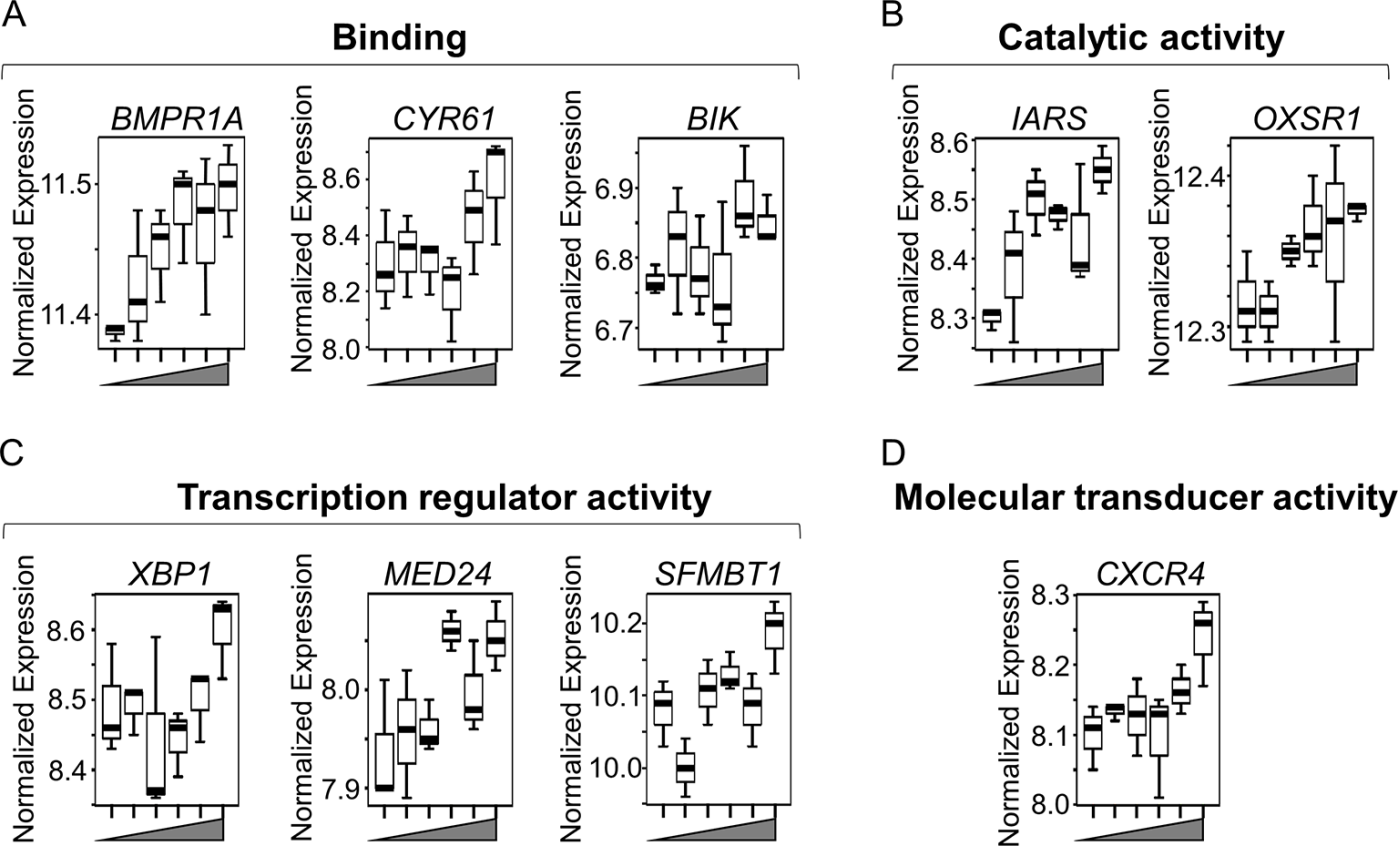
Key discriminatory genes plotted from significantly enriched gene sets altered by Mn dose. **(A–D)** Normalized expression of the representative genes (from **Figures 3B–H**), with respect to increasing Mn concentration (from left to right, 0, 1, 5, 10, 50, 100 µM), are plotted as box plots. Genes distributed in four molecular functional categories as defined by gene ontology and include **(A)** binding (GO:0005488, GO:0046982)—*BMPR1A* (bone morphogenetic protein receptor, type IA), *CYR61* (cysteine-rich, angiogenic inducer, 61), and *BIK* (BCL-2 interacting killer). **(B)** Catalytic activity (GO:0003824)—*IARS* (isoleucyl-tRNA synthase) and *OXSR1* (oxidative stress-responsive 1). **(C)** Transcription regulator activity (GO:0140110)—*XBP1* (X-box-binding protein 1), *MED24* (mediator complex subunit 24), and S*FMBT1* (Scm-like with four mbt domains 1). **(D)** Molecular transducer activity (GO:0060089)—*CXCR4* (C-X-C chemokine receptor type 4). All of the genes increased in a Mn dose-dependent manner, while apoptosis-related genes such as CYR61 and BIK showed an abrupt increase at ≥ 50 µM Mn treatment. (*P* < 0.05, *n* = 3 per Mn treatment group).

Box plots for the discriminatory genes contributing to the leading edge of the aforementioned seven enriched gene sets, which were also significant by lmreg analysis (*P* < 0.05; see **Supplementary Table S1**), are plotted in **Figures 4A–D**. All of these genes increased with Mn in a dose-dependent manner and belonged to multiple molecular functional categories highlighted in **Figure 1A**. Of these, three genes represent binding functional category (GO:0005488, GO:0046982) (**Figure 4A**)—*BMPR1A* (bone morphogenetic protein receptor 1A), which encodes a protein with neurotrophic effects involved with transmitting chemical signals from the cell membrane to the nucleus and has been suggested as a therapeutic target in Parkinson’s disease (O’Keeffe et al., 2017); *CYR61* (cysteine-rich angiogenic inducer 61), which encodes a matricellular protein which plays an important role in cell adhesion and migration, wherein *CYR61* gene induction is necessary for neuronal cell death (Kim et al., 2003); and *BIK* (BCL2-interacting killer), apoptosis-inducing gene. Two genes represented the catalytic activity category (GO:0003824) (**Figure 4B**)—*IARS* (isoleucyl-tRNA synthetase), involved in amino acid metabolism, and mutations in this gene have been associated with intellectual disability (Kopajtich et al., 2016), and *OXSR1* (oxidative stress–responsive 1), which belongs to Ser/Thr protein kinase family which regulates downstream kinases in response to environmental stress and is overexpressed in schizophrenia (Arion and Lewis, 2011). Three genes represented transcription regulator activity (GO:0140110) (**Figure 4C**)—*XBP1* (X-box-binding protein 1), transcription factor that regulates accumulation of unfolded protein in the cell and plays a role in memory and cognition (Cisse et al., 2017); *MED24* (mediator complex subunit 24), gene associated with the transcriptional coactivator complex responsible for the expression of a number of genes involved in metabolism and other cellular functions; and *SFMBT1* (Scm-like with four mbt domains 1), which regulates epigenetic silencing by association with transcriptional corepressor complexes (Lin et al., 2013). *CXCR4* (C-X-C chemokine receptor type 4), present in the molecular transducer activity category (GO:0060089) (**Figure 4D**), functions in signaling and modulating synaptic function and neuronal survival in the mature brain (Nash and Meucci, 2014). Of these, IARS was also present in the top 30 ranked genes, listed based on the VIP scores generated by PLS-DA analysis (See **Figure 2B**). Interestingly, neuronal death–associated genes such as *CYR61* and *BIK* were upregulated at 50- and 100-μM Mn exposure for 5 h, a time point at which no apparent cell death occurred but resulted in subsequent cell death at 24 h (toxic). The gene expression for *CYR61* and *BIK*, however, did not change up to 10-μM Mn exposure for 5 h (physiologic).

### Adaptive Cellular Responses Elicited by Mn at the Transcriptomic Level

**Figure 5:**
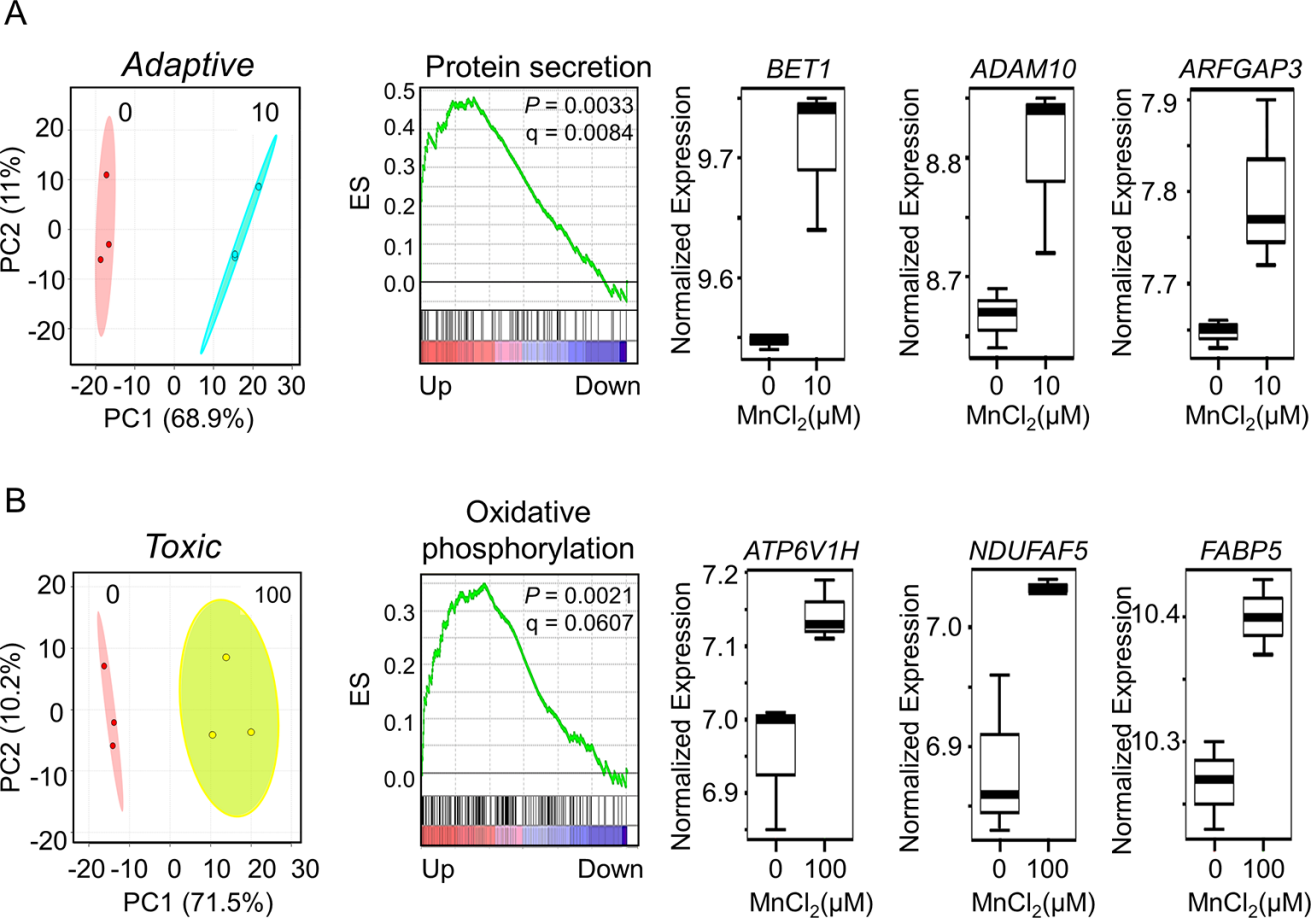
Gene set enrichment and key discriminatory genes representative of adaptive *versus* toxicological Mn content. **(A)** PLS-DA score plot depicts separation between control and 10-μM Mn-treated groups along principal component 1, which accounts for a 68.9% of the variation in adaptive Mn concentration (left panel). Enrichment plot with enrichment score (ES), *P*-value, and q-value for FDR is shown for protein secretion pathway altered by adaptive Mn concentration (middle panel). Representative genes accounting for protein secretion pathway enrichment include *BET1* (Golgi vesicular membrane-trafficking protein), *ADAM10* (metallopeptidase domain 10), and *ARFGAP3* (ADP ribosylation factor GTPase-activating protein 3) and are represented by box plots (right panel). **(B)** PLS-DA score plot shows separation between control and 100-μM Mn-treated groups along principal component 1, which accounts for a 71.5% of the variation in toxic Mn concentration (left panel). Enrichment plot is shown for oxidative phosphorylation pathway enriched by toxic Mn concentration (middle panel). Representative genes accounting for enrichment of oxidative phosphorylation pathway include *ATP6V1H* (ATPase H+ transporting V1 subunit H), *NDUFAF5* (NADH: ubiquinone oxidoreductase complex assembly factor 5), and *FABP5* (fatty acid–binding protein 5) and are shown as box plots (right panel). Details on the leading edge genes and their respective gene sets for adaptive and toxic Mn response are provided in **Supplementary Table S3** and **S4**, respectively. (*P* ≤ 0.05, n = 3 per Mn treatment group).

To discriminate potentially adaptive changes under non-toxic conditions (10 µM Mn *vs*. control) from toxic condition (100 µM Mn *vs*. control), expression data under the two Mn doses were compared by LIMMA. Results showed that 541 genes significantly differed between 10 µM Mn and control at *P* < 0.05, and PCA plots showed that these genes separated the two groups mainly along PC1 (68.9%) (**Figure 5A**, left panel). A total of eight genes from this list were common with the lmreg analysis list from **Figure 1A**, four of which were also present in the top-ranked 30 genes based on their VIP score from **Figure 2B**. For GSEA, the entire 15,066 data set were ranked according to signal to noise ratio (default GSEA) for the two Mn groups and these pre-ranked datasets were used as input for GSEA v2.0 (Subramanian et al., 2005). Alternate ranking method using *P*-value with fold change direction, as previously suggested (Xiao et al., 2014), was applied to the current data and resulted in similar results (data not shown). Treatment at 10 µM Mn resulted in enrichment of protein secretion pathway (NES = 1.83, *P*-value = 0.0033, FDR q-value = 0.0084) (**Figure 5A**, middle panel). The leading edge genes involved in this pathway include mostly Golgi residing and/or trafficking proteins such as *BET1* (Golgi vesicular membrane-trafficking protein, *P* = 0.0155), *ADAM10* (metallopeptidase domain 10, *P* = 0.0417), and *ARFGAP3* (ADP ribosylation factor GTPase-activating protein 3, *P* = 0.0359), all of which increased with 10 µM Mn (**Figure 5A**, right panel). Details on the leading edge genes contributing to the enrichment of protein secretion pathway as an adaptive response to physiological Mn dose are provided in **Supplementary Table S3**.

The same analysis comparing 100 µM Mn and control showed that 376 genes differed at *P* < 0.05. PCA plots showed that these genes separated the two groups mainly along principal component 1 (71.5%) (**Figure 5B**, left panel). A total of 142 genes from this list were common with the lmreg analysis list from **Figure 1A**, 21 of which were also present in the top-ranked 30 genes based on their VIP score from **Figure 2B**. GSEA pre-ranked analysis results showed that these differentially expressed 376 genes were mostly enriched in oxidative phosphorylation pathway (NES = 1.54, *P*-value = 0.0021, FDR *q*-value = 0.0607) (**Figure 5B**, middle panel). The leading edge genes involved in this pathway include *ATP6V1H* (ATPase H+ transporting V1 subunit H, *P* = 0.016), *NDUFAF5* (NADH: ubiquinone oxidoreductase complex assembly factor 5, *P* = 0.0313), and *FABP5* (fatty acid–binding protein 5, *P* = 0.0411), all of which increased with 100 µM Mn (**Figure 5B**, right panel). Also, mitochondrial genes that were enriched in this pathway, but did not reach the cutoff value of *P* < 0.05, were *MDH2* (mitochondrial malate dehydrogenase, *P* = 0.0506) and outer mitochondrial membrane protein *TOMM22* (translocase of outer mitochondrial membrane 22, *P* = 0.1164), which increased with 100 µM Mn. Details on the leading edge genes contributing to the enrichment of oxidative phosphorylation pathway as a toxic Mn dose response are provided in **Supplementary Table S4**. Protein secretion–related genes (*BET1*, *ADAM10*, and *ARFGAP3*) that significantly increased at physiological Mn dose showed a declining trend at toxic Mn dose. Oxidative phosphorylation–related genes (*ATP6V1H*, *NDUFAF5*, and *FABP5*) showed Mn dose-dependent increase, which was significant at toxic Mn dose but not significant at physiological Mn dose (**Supplementary Figure 1**). The results thus show that changes in the Golgi-associated genes that function in protein secretion occur in response to Mn that does not cause cell death (adaptive) while mitochondria-associated genes that function in energy metabolism respond to Mn concentration that causes subsequent cell death (toxic).

**Figure 6:**
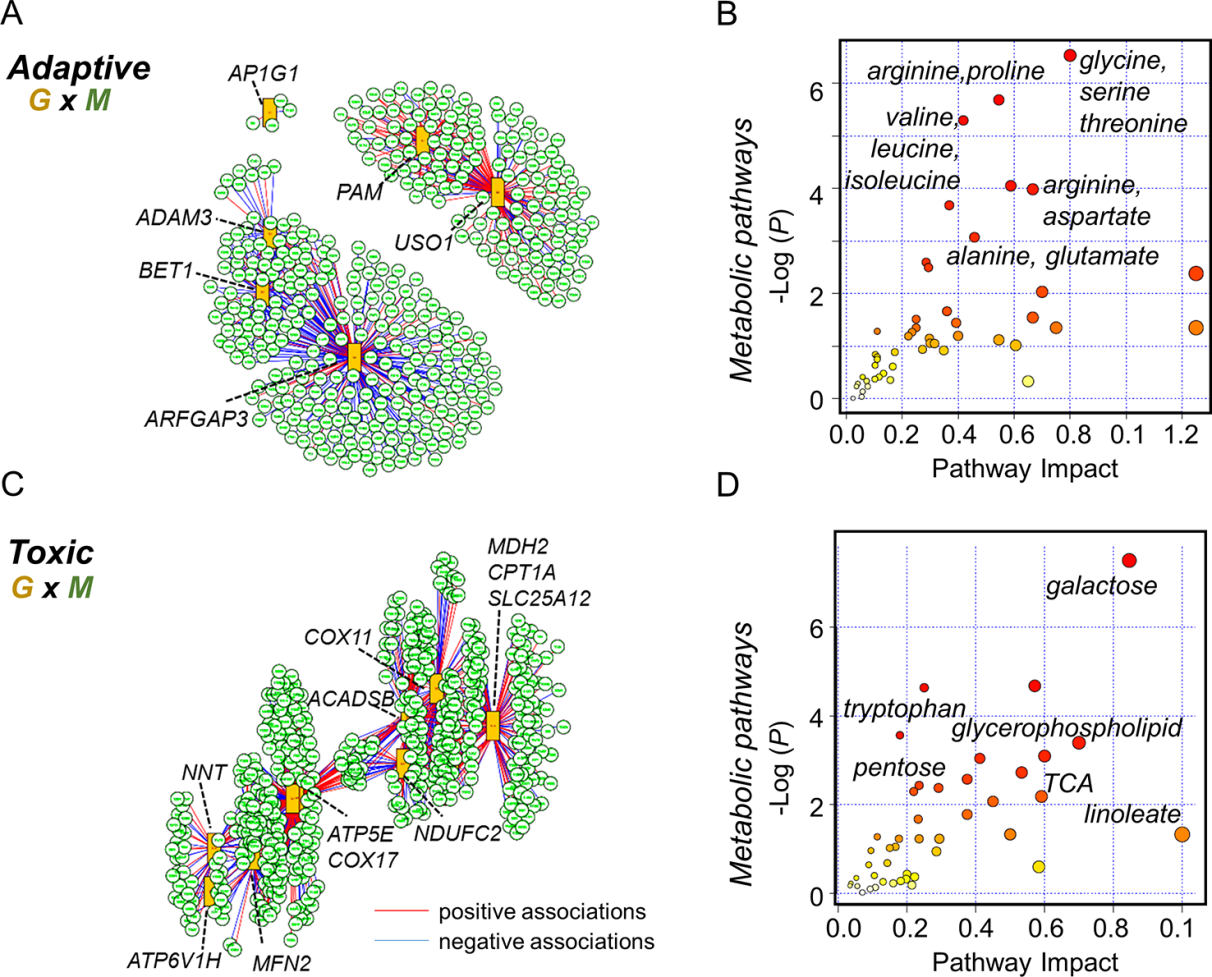
Integrated transcriptome-metabolome wide association study (TMWAS) of Mn-dependent adaptive and toxic gene response pathways. **(A)** *xMWAS* integration analysis using metabolites and GSEA protein secretion gene set, as an adaptive response to 10 μM Mn, with 0.4 correlation threshold at *P* < 0.05 with sPLS canonical correlation method. **(B)** The protein secretion genes were associated with a number of amino acid metabolic pathways, determined by gene-metabolite joint analysis using MetaboAnalyst 4.0. Glycine, serine, and threonine metabolic pathway was the top most pathway associated with protein secretion gene set at 10 μM Mn. **(C)** Using similar parameters, metabolite network structure was obtained for GSEA oxidative phosphorylation gene set, as a toxic response to 100 μM Mn. In this, two of the genes (*NDUFAF5*, *FABP5)* shown in **Figure 5B** were not hubs but were correlated with hub genes, *NDUFC2* (NADH:ubiquinone oxidoreductase subunit C2), and *ACADSB* (short-chain acyl-CoA dehydrogenase), *CPT1A* (carnitine palmitoyltransferase 1A), respectively. **(D)** The oxidative phosphorylation genes were associated with energy, lipid, and neurotransmitter pathways, determined by joint analysis. Galactose metabolic pathway was the top most pathway associated with oxidative phosphorylation gene set at 100 μM Mn followed by glycerophospholipid and tryptophan metabolism. Red lines represent positive correlation, and blue represents negative correlation between genes (G: orange rectangles) and metabolites (M: green circle). Metabolites associated with specific genes are provided in the text. Details on the leading edge genes and their respective gene sets for adaptive and toxic Mn response used in data integration are provided in **Supplementary Tables S3** and **S4**, respectively.

Multi-omics offers the opportunity to understand the flow of information that underlies disease (Hasin et al., 2017), and new differential network analysis tools provide improved sensitivity to identify changes in the interplay between molecules rather than changes in single molecules (Lichtblau et al., 2017). Accordingly, we performed integrated analysis for the present gene expression data, with previously reported untargeted metabolomics data (Fernandes et al., 2019). We specifically used *xMWAS* 0.54 (Uppal et al., 2018) with targeted gene expression data for the protein secretion gene set at 10 μM Mn to determine top transcriptome-metabolome associations for the physiologic Mn response (**Figure 6**). The results show that a correlation network analysis using an input of 31 protein secretion genes (**Supplementary Table S3**) and 6296 metabolic features resulted in 400 significantly associated metabolites. The tentative KEGG IDs for the associated metabolites obtained from *mummichog* software 1.0.9 (Li et al., 2013) and list of gene names in the protein secretion gene set (See **Supplementary Table S3**) were used as input in joint pathway analysis using MetaboAnalyst 4.0 (Chong et al., 2018). Over-representation analysis was based on hypergeometric analysis method to identify enriched metabolic pathways and is plotted as –log of *P*-value on the x-axis (**Figure 6A**). The most significant metabolic pathway associated with protein secretion gene set was glycine, serine, and threonine pathway (*P*-value < 0.001, FDR = 0.11, pathway impact 0.8) followed by a number of amino acid–related pathways as shown in **Figure 6B**. Annotated metabolites from the enriched metabolic pathways include lysine (M+Na, *m/z* 169.0950), alanine (M+H, *m/z* 90.0551), and trans-4-hydroxy proline (M+H, *m/z* 132.0661). Using the same untargeted metabolomics dataset with 6,296 features, the integrative analysis was performed with oxidative phosphorylation gene set (75 genes, **Supplementary Table S4**), enriched as a toxic response to 100 μM Mn, to reveal associated metabolic pathways and metabolites (**Figure 6C**). A total of 496 metabolites were associated with oxidative phosphorylation gene set. The most significant metabolic pathway associated with oxidative phosphorylation gene set was galactose metabolic pathway (*P*-value < 0.001, FDR = 0.04, pathway impact 0.85) followed by a number of lipid, neurotransmitter, and energy-related pathways such as glycerophospholipid, tryptophan, and TCA metabolism (**Figure 6D**). Annotated metabolites from the enriched pathways include nicotinamide (M+H, *m/z* 123.0554), linoleate (M+H, *m/z* 281.2478), glucosylceramide (M+H, *m/z* 644.5087), and dopamine sulphate (M+Na, *m/z* 256.0253).

## Discussion

The present TWAS results of Mn dose response in SH-SY5Y cells demonstrate that transcripts of the protein secretory pathway of the ER-Golgi complex are upregulated at a concentration that does not cause subsequent cell death while transcripts for mitochondrial proteins are upregulated at a concentration that is associated with mitochondrial dysfunction, energy failure, and subsequent cell death. These results are consistent with previous findings using synchrotron X-ray fluorescence nanoimaging in dopaminergic cells which showed that Mn is located principally within the Golgi apparatus compared to other organelles under physiological conditions (Carmona et al., 2010). The study showed that, under cytotoxic Mn concentrations or when the Golgi apparatus is collapsed, Mn is redistributed to other subcellular compartments. Consequently, Mn toxicity could occur when the Golgi storage capacity is exceeded and results in increased Mn in critical targets such as the mitochondria (Carmona et al., 2010).

The present results show that Mn increased gene expression for multiple Golgi-residing proteins with 10 μM Mn (physiological), including *BET1*, *ADAM10*, and *ARFGAP3*, all of which play a central role in the protein secretion pathway (See **Figure 5**). The Golgi complex is an integral component of the secretory pathway and is responsible for receiving newly synthesized proteins from the ER, modifying the proteins and subsequently exporting these proteins to either the endosomal-lysosomal system or the cell surface. During protein trafficking through the Golgi complex, key protein modifications are made, including oxidation, phosphorylation, glycosylation, and proteolytic cleavage (Bexiga and Simpson, 2013). In the mammalian cells, Mn ions are required for the addition of complex carbohydrates onto glycosylated proteins in the lumen of the Golgi complex (Kaufman et al., 1994). Accumulation of Mn in the secretory pathway has also been proposed as an important detoxification pathway for excess Mn within the cell (Varki, 1998; Van Baelen et al., 2004). SNARE (soluble N-ethylmaleimide-sensitive factor attachment protein receptor) proteins are responsible for fusion of vesicles within the cell and with the plasma membrane, to facilitate the release of the content of the vesicle in the cell and/or to the extracellular environment (Han et al., 2017). BET1, in particular, is responsible for ER-Golgi vesicular transport and is a member of the SNARE complex. As such, upregulation of *BET1* gene expression with 10 μM Mn suggests a potential role for the secretory pathway in sequestering Mn and maintaining cellular homeostasis.

ADAM10, a disintegrin metallopeptidase, also resides on the Golgi complex. In neurons, ADAM10 has α-secretase activity that functions in proteolytic processing of the amyloid precursor protein (Haass et al., 2012). ADAM10 overexpression in Alzheimer’s disease mouse model prevents formation of amyloid plaques and is a therapeutic target for Alzheimer’s disease (Postina et al., 2004). *ADAM10* knockout is embryonic lethal in mice due to function in establishment of the brain cortex (Jorissen et al., 2010). Our data showing increase in *ADAM10* gene expression with increased physiological Mn therefore suggests Mn-dependent adaptive response wherein ADAM10 (as putative alpha-secretase) precludes toxic amyloid-beta-peptide accumulation.

ARFGAP3 is an ADP ribosylation factor in the early protein secretory pathway, which is required for the dissociation of coat proteins from Golgi-derived membranes and vesicles, to allow for fusion of these vesicles with target compartments (Kartberg et al., 2010). ARFGAP3 is a component of the COPI (coat protein complex I) pathway mediating the recycling of proteins between ER and the Golgi (Kartberg et al., 2010). Mn-dependent increase in *ARFGAP3* gene expression therefore could represent activation of the early secretory pathway promoting increased protein trafficking to keep up with the cellular demand. Together, these results suggest upregulation of *BET1*, *ADAM10*, and *ARFGAP3* gene expression that could be an adaptive mechanism and support targeted studies *in vivo* to determine the therapeutic value of targeting these pathways for neuroprotection.

Previously microarray analysis of human SH-SY5Y cells in response to 100 μM Mn for 30 days demonstrated upregulation of apoptotic pathways which was interpreted to be a causative mechanism for Mn-dependent chronic neuronal toxicity (Gandhi et al., 2018). Although information on the intracellular Mn concentration was not available (Gandhi et al., 2018), the Mn treatment probably resulted in higher accumulation than the 5-h treatment with 100 μM Mn that we used. Because we performed measurements before cell death was observed, the transcriptomic responses do not reflect immediate cell death. In addition to apoptosis, mitochondrial dysfunction and oxidative stress have been proposed as Mn-dependent neurotoxic responses in multiple functional studies (Zhang et al., 2004; Alaimo et al., 2013; Fernandes et al., 2017; Smith et al., 2017). Our transcriptome data show that 100 μM Mn increased gene expression for multiple oxidative phosphorylation and energy-mediated processes in the human SH-SY5Y cells, including *ATP6V1H*, *NDUFAF5*, and *FABP5* prior to impending cell death (See **Figure 5**). *ATP6V1H*, identified as a gene in the oxidative phosphorylation pathway couples ATPase activity to proton flow and mediates acidification of intracellular organelles (https://www.genome.jp/kegg-bin/show_pathway?hsa00190+51606). Previously published literature has suggested *ATP6V1H* gene may play a role in the pathogenesis of neurodegenerative diseases after the discovery of SNPs in this gene associated with β-site APP cleaving enzyme in cerebrospinal fluid of Alzheimer’s patient (Hu et al., 2018). In the current study, Mn-induced upregulation of *ATP6V1H* suggests increased cellular energy requirement. NDUFAF5 is required for complex I assembly and is located in the inner mitochondrial membrane (Sugiana et al., 2008; Gerards et al., 2010; Giachin et al., 2016). Complex I activity is inhibited by MnCl_2_ in PC12 cells (Galvani et al., 1995). Hence, in the current study, toxic Mn dose response indicates increased demand for assembly of new complex I in response to Mn toxicity. FABP5 is involved in lipid metabolism and binds specifically to long-chain fatty acids to facilitate cellular transport to different organelles (Liu et al., 2010). FABP5, at the same time, also activates nuclear receptor PPAR (peroxisome proliferator-activated receptor) activity in the brain, which in turn regulates cellular energy metabolism (Wang, 2010). Increased FABP5 is observed under conditions of injured and regenerating neurons where it plays a critical role in neuronal differentiation (Liu et al., 2008; Liu et al., 2010) and also participates in regulating cognitive function (Yu et al., 2014). Thus, within the context of existing literature, our results show that Mn-induced toxic responses are governed by the energy demand supported by mitochondria.

Studies (Cheng et al., 2018) have shown chronic MnSO_4_ exposure in rats, by intraperitoneal injection for 24 weeks, resulted in Mn dose-dependent increase in stress-responsive genes such as Hsp70, accompanied with changes in PI3/AKT signaling events, in the rat hippocampus. Their data suggest that MMT (methylcyclopentadienyl manganese tricarbonyl used in gasoline) combustion product such as MnSO_4_ could result in alteration in signaling events of central nerve cells and that heat-shock proteins could serve as a biomarker of metal toxicity (Cheng et al., 2018). Similarly, in our *in vitro* study, we observed HSPA9 (mitochondrial HSP70 protein 9) and HSPA5 (endoplasmic reticulum HSP70 protein 5) enriched in the MTORC1 signaling and UPR pathway (See **Supplementary Table S2**). HSPA9 was also enriched in mitochondrial oxidative phosphorylation pathways with toxicological Mn dose (See **Supplementary Table S4**). Also, HSP70 and HSP40 (DnaJ) functionally interact with soluble mutant huntingtin oligomers suppressing unfolded protein toxicity (Lotz et al., 2010) and thus protect against neurodegenerative disease (Zarouchlioti et al., 2018). In the current study, DnaJB11 (HSP40) gene expression significantly declined with Mn dose (See **Supplementary Table S1**), which suggest increased propensity of unfolded protein accumulation with increased Mn dose. Thus, our results using an *in vitro* Mn toxicity model supports previously published studies that suggest heat-shock proteins as biomarkers of metal toxicity.

In addition to heat-shock proteins, C-X-C chemokine receptor type 4 (CXCR-4) is implicated in several Mn-regulated biological processes such as glycolysis, hypoxia, and mTORC-1 signaling (See **Figure 3**). Toxic Mn exposure in the current study results in an increased CXCR4 expression. CXCR4 expression is increased in human neurodegenerative diseases and in mouse models of tauopathies (Bonham et al., 2018). CXCR4 regulates a number of cellular processes such as apoptosis, cell cycle, chemotaxis-neuronal guidance, proliferation, transcription, calcium homeostasis, and PI3K/AKT signaling (Bezzi et al., 2001; Khan et al., 2003; Yang et al., 2013; Merino et al., 2016; Cheng et al., 2017). Published reports demonstrate that CXCR4 mRNA expression could be regulated by metal alloys (Drynda et al., 2015), calcium (Wu et al., 2009), and cAMP (Liang et al., 2015; Busillo and Benovic, 2007). Metal homeostasis, calcium, and cAMP are regulated by Mn levels in the cell (Streifel et al., 2013; Ducic et al., 2015). Hence, our data in the context of existing literature suggest that Mn exposure within the cells could be a result of these secondary messengers resulting in increased CXCR4 expression, which in turn could regulate multiple downstream cellular processes associated with neuronal disorders.

The present research follows the recommendations for toxicity testing in the 21st century to use of human cells, dose-response modeling, and computational and bioinformatics approaches to improve strategies to protect human health (Council, 2007; Boekelheide and Campion, 2010; Krewski et al., 2010). The research shows that selection of doses without subsequent toxicity and doses with subsequent toxicity has the potential to distinguish adaptive mechanisms from those that perturb the toxicity pathways (Krewski et al., 2010). Importantly, the current results provide evidence for protective responses involving the secretory pathway (See **Figure 6B**) not evident from the results seen in metabolome-wide association study showing changes in neuroprotective amino acids (Fernandes et al., 2019). However, gene expression under toxic conditions showed concordant effects with those expected from impaired mitochondrial respiration (Fernandes et al., 2017; Fernandes et al., 2019).

Several limitations of the present study are recognized. Studies involve a single human cell line that cannot replicate the complex physiologic and toxic responses of intact brain. Thus, *in vivo*–based systems biology approaches will be needed to test whether upregulation of protein secretion pathway and deficit in mitochondrial function can be used to improve assessment and management of risks of Mn neurotoxicity. The present data, along with the extensive previous use of human SH-SY5Y neuroblastoma cells as a model system for study of neurological disorders (Oguchi et al., 2017; Song and Kim, 2017; Yao et al., 2018), provide a foundation for such research. A second limitation is the use of only one omics technology to interpret adaptive from toxic Mn cellular responses. Transcriptome changes do not always reflect proteomic, metabolomics, or functional outcomes. Our previous studies with this cell model show, however, that functional changes of mitochondria and pathways of energy metabolism occur over the same adaptive and toxic Mn concentration ranges (Fernandes et al., 2017; Fernandes et al., 2019). Additionally, in **Figure 6**, we have attempted to integrate metabolomics data with targeted enriched gene sets to demonstrate metabolic changes that are associated with transcriptomic alterations observed either at adaptive or toxic Mn dose. Nonetheless, further integrated omics and time-course studies will be needed to improve discrimination of physiological mechanisms to manage Mn availability from those toxicological mechanisms by which Mn causes neuropathology.

## Conclusion

**Figure 7:**
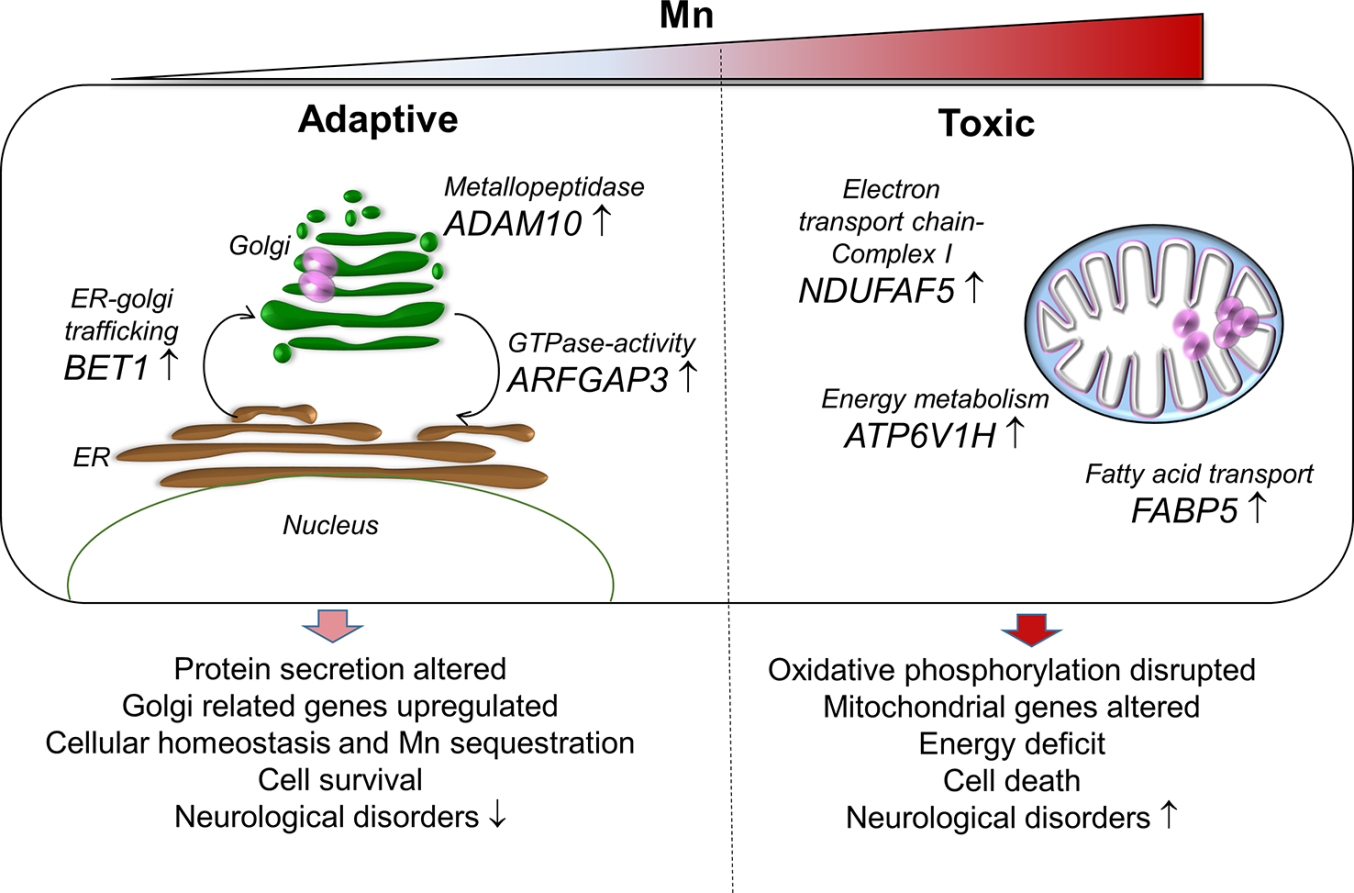
A proposed schematic representation of Mn-dependent adaptive and toxic response pathways. Adaptive (left), human neuronal cells (SH-SY5Y) exposed to physiological Mn resulted in enrichment of protein secretion pathway by increasing gene expression for Golgi-residing proteins—*BET1*, *ADAM10*, and *ARFGAP3*. *BET1* participates in vesicular transport from the endoplasmic reticulum (ER) to the Golgi complex thereby participating in protein transport and processing. *ADAM10* has α-secretase activity in the neurons for proteolytic processing of the amyloid precursor proteins, thereby preventing accumulation of unwanted proteins. *ARFGAP3* is a GTPase-activating protein that regulates the early secretory pathway of proteins. The protein secretion pathway plays an important role in Mn sequestration and maintaining cellular homeostasis, thus promoting cell survival that could protect against neurological diseases. Toxic (right), cells exposed to toxic Mn disrupts energy metabolism by enrichment in oxidative phosphorylation pathway involving genes that participate in energy metabolism—*ATP6V1H*, *NDUFAF5*, and *FABP5*. *ATP6V1H* is a gene in the oxidative phosphorylation pathway that couples ATPase activity to proton flow and mediates acidification of intracellular organelles. *NDUFAF5* is a gene that is extremely essential in complex I assembly, which is required for the function of the mitochondrial electron transport chain. FABP5 is gene that regulates lipid metabolism by binding to long-chain fatty acids specifically and transporting them. Disruption in cellular and mitochondrial energy metabolism along with oxidative stress could lead to impending cell death and inability to recover. This combination could eventually be detrimental and result in Mn toxicity and neurological diseases.

In conclusion, the current study using RNA-Seq with an *in vitro* cellular model to examine transcriptomic response to Mn dose provided detailed insight into potential mechanisms for adaptation to Mn through ER-Golgi-mediated gene responses and failed mechanisms to protect against mitochondrial energy disruption (proposed schematic representation, **Figure 7**). Importantly, the transcriptome responses at toxic Mn dose demonstrated patterns observed with neurological diseases and suggest that differential functions of the secretory pathway and mitochondria could provide a basis to improve detection and management of adverse environmental and occupational Mn exposures.

## Data Availability

The datasets generated for this study can be found in NCBI’s Gene Expression Omnibus (GEO), GEO accession GSE129336, https://www.ncbi.nlm.nih.gov/geo/query/acc.cgi?acc=GSE129336.

## Author Contributions

DJ, Y-MG, and JF participated in the study design and manuscript preparation; JF, JC, and LH conducted research; LL and JF processed RNA-Seq data files through the bioinformatics pipeline. JF performed RNA-Seq data analysis, gene set enrichment, pathway analysis, interpreted data, and wrote the first draft of the manuscript; JF and KU performed statistical and bioinformatics analysis of the RNA-Seq data. JC and XH provided assistance with gene set enrichment analysis. JF, JC, LL, KU, XH, Y-MG and DJ reviewed the figures. DJ and Y-MG had primary responsibility for final content. All authors read and approved the final version of the manuscript.

## Funding

This work was funded by NIEHS Grants R01 ES023485 (DJ and Y-MG), R21 ES025632 (DJ and Y-MG), P30 ES019776 (DJ), and NIH S10 OD018006 (DJ).

## Conflict of Interest Statement

The authors declare that the research was conducted in the absence of any commercial or financial relationships that could be construed as a potential conflict of interest.
