# Supplementary material for: Transcriptome Analysis Reveals Distinct Responses to Physiologic versus Toxic Manganese Exposure in Human Neuroblastoma Cells
Source: Front Genet. 2019 Jul 24;10:676. doi: 10.3389/fgene.2019.00676 (PMC6668488; doi:10.3389/fgene.2019.00676)
Supplement: Supplementary file 5 [file Presentation_1.pptx]

## Slide 1
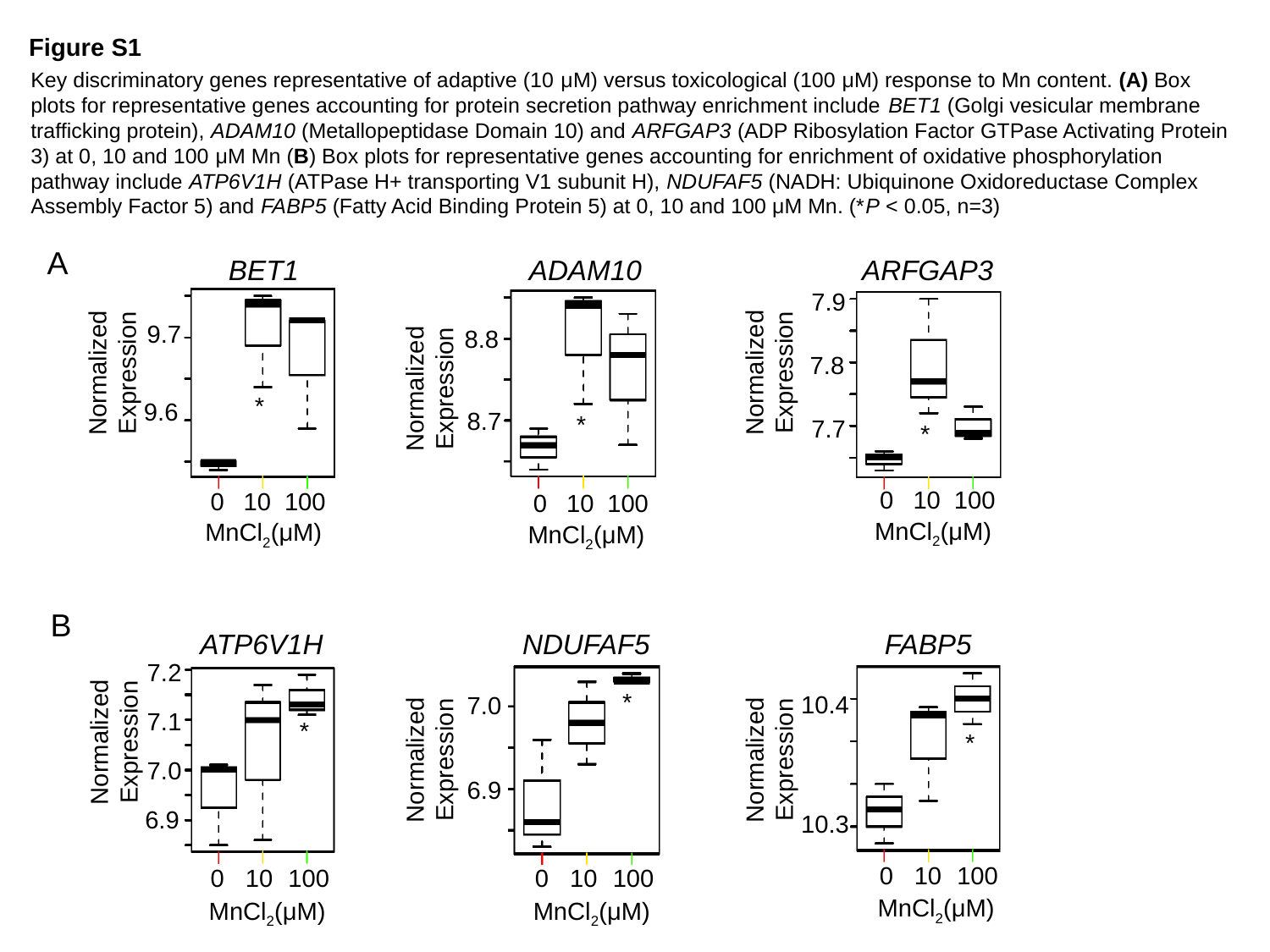

Figure S1
Key discriminatory genes representative of adaptive (10 μM) versus toxicological (100 μM) response to Mn content. (A) Box plots for representative genes accounting for protein secretion pathway enrichment include BET1 (Golgi vesicular membrane trafficking protein), ADAM10 (Metallopeptidase Domain 10) and ARFGAP3 (ADP Ribosylation Factor GTPase Activating Protein 3) at 0, 10 and 100 μM Mn (B) Box plots for representative genes accounting for enrichment of oxidative phosphorylation pathway include ATP6V1H (ATPase H+ transporting V1 subunit H), NDUFAF5 (NADH: Ubiquinone Oxidoreductase Complex Assembly Factor 5) and FABP5 (Fatty Acid Binding Protein 5) at 0, 10 and 100 μM Mn. (*P < 0.05, n=3)
A
ARFGAP3
BET1
ADAM10
7.9
9.7
8.8
Normalized Expression
Normalized Expression
7.8
Normalized Expression
*
9.6
8.7
*
7.7
*
0
10
100
0
10
100
0
10
100
MnCl2(μM)
MnCl2(μM)
MnCl2(μM)
B
ATP6V1H
NDUFAF5
FABP5
7.2
*
10.4
7.0
7.1
Normalized Expression
*
*
Normalized Expression
Normalized Expression
7.0
6.9
6.9
10.3
0
10
100
0
10
100
0
10
100
MnCl2(μM)
MnCl2(μM)
MnCl2(μM)
