# Supplementary material for: Transcriptome Analysis Reveals Distinct Responses to Physiologic versus Toxic Manganese Exposure in Human Neuroblastoma Cells
Source: Front Genet. 2019 Jul 24;10:676. doi: 10.3389/fgene.2019.00676 (PMC6668488; doi:10.3389/fgene.2019.00676)
Supplement: Supplementary file 6 [file DataSheet_1.pdf]

ATCC®

# CERTIFICATE OF ANALYSIS

| | |
|---|---|
| **ATCC® Number:** | **CRL-2266™** |
| **Lot Number:** | **62431864** |
| **Name:** | **SH-SY5Y** |
| **Description:** | **Neuroblastoma** |
| **Species:** | **Human (*Homo sapiens*)** |
| **Volume/Ampule:** | **Approximately 1 mL** |
| **Date Frozen:** | **January 19, 2015** |
| **Recovery:** | **A T-25 setup at a dilution of 1:10 reaches approximately 20-30% confluence in 2 days.** |
| | **A T-75 setup at a dilution of 1:15 reaches approximately 20% confluence in 3 days.** |
| **Product Format:** | **Cells cryopreserved in the appropriate cryopreservation medium** |
| **Expiration Date:** | **Not applicable** |
| **Storage Conditions:** | **Vapor phase of liquid nitrogen** |

| Test / Method | Specification | Result |
|---|---|---|
| **Ampule passage number** | Report results | 27 |
| **Population doubling level (PDL)** | Report results | Not applicable |
| **Total cells/ampule**<br>(Cell count using Trypan Blue stain method) | Report results | $1.7 \times 10^6$ total cells/ampule |
| **Post-freeze viability**<br>(Cell count using Trypan Blue stain method) | ≥ 50.0% | 74.6% |
| **Growth properties**<br>(Visual observation method) | Mixed – adherent and/or suspension | Mixed – adherent and suspension |
| **Morphology**<br>(Visual observation method) | Neuronal-like | Neuronal-like |
| **Test for mycoplasma contamination**<br>Hoechst DNA stain (indirect) method<br>Agar culture (direct) method | <br>None detected<br>None detected | <br>None detected<br>None detected |
| **Species determination: COI assay (interspecies)** | Human | Human |

**ATCC**
10801 University Boulevard
Manassas, VA 20110-2209 USA
www.atcc.org

800-638-6597 or 703-365-2700
Fax: 703-365-2750
E-mail: tech@atcc.org
or contact your local distributor

ATCC

# CERTIFICATE OF ANALYSIS

**ATCC® Number:** **CRL-2266™**

**Lot Number:** **62431864**

| | | |
|---|---|---|
| **Species determination: STR analysis (intraspecies)** | **Human (Unique DNA Profile)**<br>TH01: 7, 10<br>D5S818: 12<br>D13S317: 11<br>D7S820: 7, 10<br>D16S539: 8, 13<br>CSF1PO: 11<br>Amelogenin: X<br>vWA: 14, 18<br>TPOX: 8, 11 | **Human (Unique DNA Profile)**<br>TH01: 7, 10<br>D5S818: 12<br>D13S317: 11<br>D7S820: 7, 10<br>D16S539: 8, 13<br>CSF1PO: 11<br>Amelogenin: X<br>vWA: 14, 18<br>TPOX: 8, 11 |
| **Sterility test (BacT/ALERT 3D)**<br>iAST bottle (aerobic) at 32°C<br>iNST bottle (anaerobic) at 32°C | <br>No growth<br>No growth | <br>No growth<br>No growth |
| **Human pathogenic virus testing**<br>(PCR-based assay for HIV, HepB, HPV, EBV, and CMV) | Report results | HIV – None detected<br>HepB – None detected<br>HPV – None detected<br>EBV – None detected<br>CMV – None detected |

jsalisbury@atcc.org

Digitally signed by jsalisbury@atcc.org
DN: cn=jsalisbury@atcc.org
Date: 2015.04.24 13:12:43 -04'00'

Quality Assurance Specialist; Quality Assurance

ATCC hereby represents and warrants that the material provided under this certificate is pure and has been subjected to the tests and procedures specified and that the results described, along with any other data provided in this certificate, are true and correct to the best of the company's knowledge and belief. This certificate does not extend to the growth and/or passage of any living organism or cell line beyond what is supplied within the container received from ATCC.

This product is intended to be used for laboratory research use only. It is not intended for use in humans, animals, or for diagnostics. Appropriate Biosafety Level (BSL) practices should always be used with this material. Refer to the Product Information Sheet for instructions on the correct use of this product.

ATCC products may not be resold, modified for resale, used to provide commercial services, or to manufacture commercial products without prior written agreement from ATCC.

© 2014 American Type Culture Collection. The ATCC trademark and trade name are owned by the American Type Culture Collection.

**ATCC**
10801 University Boulevard
Manassas, VA 20110-2209 USA
www.atcc.org

800-638-6597 or 703-365-2700
Fax: 703-365-2750
E-mail: tech@atcc.org
or contact your local distributor
